# Decreased Sensitivity of the Serological Detection of Feline Immunodeficiency Virus Infection Potentially Due to Imported Genetic Variants

**DOI:** 10.3390/v11080697

**Published:** 2019-07-31

**Authors:** Julia Frankenfeld, Theres Meili, Marina L. Meli, Barbara Riond, A. Katrin Helfer-Hungerbuehler, Eva Bönzli, Benita Pineroli, Regina Hofmann-Lehmann

**Affiliations:** Clinical Laboratory, Department for Clinical Diagnostics and Services, and Center for Clinical Studies, Vetsuisse Faculty, University of Zurich, Winterthurerstrasse 260, 8057 Zurich, Switzerland; jfrankenfeld@vetclinics.uzh.ch (J.F.); tmeili@vetclinics.uzh.ch (T.M.); mmeli@vetclinics.uzh.ch (M.L.M.); briond@vetclinics.uzh.ch (B.R.); khungerbuehler@vetclinics.uzh.ch (A.K.H.-H.); eboenzli@vetclinics.uzh.ch (E.B.); bpineroli@vetclinics.uzh.ch (B.P.)

**Keywords:** Feline immunodeficiency virus, retrovirus, lentivirus, domestic cat, serology, point-of-care test, Western blot, gold standard, veterinary sciences

## Abstract

Feline immunodeficiency virus (FIV) is a lentivirus of domestic cats worldwide. Diagnosis usually relies on antibody screening by point-of-care tests (POCT), e.g., by enzyme-linked immunosorbent assays (ELISA), and confirmation using Western blot (WB). We increasingly observed ELISA-negative, WB-positive samples and aimed to substantiate these observations using 1194 serum/plasma samples collected from 1998 to 2019 primarily from FIV-suspect cats. While 441 samples tested positive and 375 tested negative by ELISA and WB, 81 samples had discordant results: 70 were false ELISA-negative (WB-positive) and 11 were false ELISA-positive (WB-negative); 297 ambiguous results were not analyzed further. The diagnostic sensitivity and specificity of the ELISA (82% and 91%, respectively) were lower than those reported in 1995 (98% and 97%, respectively). The diagnostic efficiency was reduced from 97% to 86%. False ELISA-negative samples originated mainly (54%) from Switzerland (1995: 0%). Sixty-four false ELISA-negative samples were available for POCT (SNAP^TM^/WITNESS^R^): five were POCT-positive. FIV RT-PCR was positive for two of these samples and was weakly positive for two ELISA- and POCT-negative samples. Low viral loads prohibited sequencing. Our results suggest that FIV diagnosis has become more challenging, probably due to increasing travel by cats and the introduction of new FIV isolates not recognized by screening assays.

## 1. Introduction

Feline immunodeficiency virus (FIV) is a lentivirus with a worldwide distribution [1,2,3]. Infection with this retrovirus can lead to immunodeficiency in domestic cats [1,2,4] while the pathogenic potential in wild felids is less well known [3,5,6,7,8,9]. Due to a lack of proofreading capacity of the reverse transcriptase, there is a high error rate during replication and low replicative fidelity [10,11,12,13]. Currently, five major clades or subtypes of FIV (A–E) have been described based on phylogenetic analyses of the variable regions 3–5 of the FIV *env* gene as well as a portion of the *gag* gene [14,15,16,17,18]. While there is some genetic intraclade diversity, the genetic distance between different FIV clades was found to be more than 17% [14,15,17]. Recently, strains were tentatively assigned to new clades in Brazil, Turkey, the USA, Portugal, and New Zealand, of which the two latter clusters exhibit the new subtypes F and U-NZ*env* [19,20,21,22,23,24].

FIV clade A strains are found worldwide [16,25,26,27,28]; the other clades show varying geographic prevalence, and the separate evolution of these clades in geographical distinct areas [14,29] and introduction into other areas has been proposed [15,26,30,31,32,33]. The most prevalent FIV clades found in Europe are A and B, with clade A being predominant in Northern Europe (Germany, Benelux, and the UK) [16,32,34,35,36] and clade B occurring more frequently in Southern Europe (Portugal, Italy, Austria, Croatia, and Turkey) [16,20,24,29,34,37]. In North America, FIV clades A, B, and C have been described [15,16,31,33]. In South America, clades A, B, and E have been reported, with clades B and E being predominant and clade E only being described in this geographic area so far [18,28,38,39,40,41]. Limited information on the FIV strains and clades is available for Asia. Clade C seems to be the most common in Taiwan and Vietnam [30,42,43]. Subtype A has been described in China [44], and subtypes A, B, C, and D have been reported in Japan. B and D were the most prevalent subtypes, and clade D was found only in Japan and Vietnam [26,30]. Clades A and B are distributed in Australia [45,46], while A, C, and U-NZ*env* are found in New Zealand [27]. Interestingly, a cat can be infected concurrently with several FIV strains [47,48,49]. Overall, the geographically restricted evolution of some subtypes, such as D, E, and F, and the increasing import of domestic cats, some of them possibly coinfected with FIV strains of more than one subtype, might result in intersubtype recombinants and changes in the locally prevailing FIV clades [16,17,26,27,30,31,32,33,34,41,42,50].

The laboratory diagnosis of FIV infection primarily relies on the detection of antibodies against FIV in infected cats, since FIV loads in the peripheral blood are usually very low and antibodies to FIV are an almost universal feature in FIV-infected cats [51,52,53,54,55,56,57]. Furthermore, genetic diversity is known to lead to challenges in the molecular diagnosis of the infection [31,54,58,59,60]. The recombination of viral strains and emerging antigenic variants might also result in antibodies that are no longer recognized by common diagnostic tests [53,61,62,63,64]. For practitioners, diagnostic point-of-care tests (POCT) that quickly detect antibodies are the method of choice [53,65,66,67,68,69]. Antibodies against the FIV transmembrane protein (TM) are the most reliable in terms of both their initial appearance post infection and their duration of detection in the blood [52,53,70,71,72]. Therefore, many POCT and enzyme-linked immunoassays (ELISA) used in diagnostic laboratories use FIV-TM as the capture antigen, but capsid protein has also been added to some tests [56,66,68,73]. The detection of FIV antibodies by Western blot (WB) is considered the gold standard and is used for the confirmation of FIV diagnosis in cases of ambiguous POCT and ELISA results [56,65,70,74,75,76,77]. In addition, WB is recommended to confirm any ELISA- and POCT-positive results in countries with a low FIV prevalence, since the positive predictive value of positive ELISA and POCT results is low under these circumstances [56,76,78].

For many years, samples from cats suspected of FIV infection (ambiguous or positive POCT results) have been sent to our laboratory for the confirmation of FIV diagnosis using Western blotting [70,74]. Furthermore, we have been receiving samples for primary FIV screening purposes with FIV-TM ELISA [53]. During routine testing, samples would be sporadically found that were negative for FIV-TM ELISA and positive for WB. This phenomenon had already been described during the development of the FIV-TM ELISA in 1995, but this was found for only four of 194 tested samples, and they were all from cats that lived outside of Switzerland [53].

We hypothesized that the number of FIV infections that are undetectable by FIV-TM ELISA and POCT has increased over the years due to an increased number of cats with a travel or import history, which has thus introduced novel FIV isolates into the sample population. Thus, the aim of this study was to systematically test samples that have been sent to our laboratory over two decades (1998–2019) using FIV-TM ELISA as well as WB, which is the gold standard for FIV testing. In cases of discrepant results with sufficient sample volumes, two commercially available POCT (SNAP^TM^ Combo Plus FeLV Ag/FIV Ab, IDEXX Laboratories, Westbrook, ME, USA; WITNESS^R^ FIV, Zoetis, Delémont, Switzerland) and RT-PCR assays [58,60,79,80] (FTvet Feline Anaemia I, Fast Track Diagnostics, Esch-sur-Alzette, Luxembourg) were performed. In addition, an attempt was made to sequence all RT-PCR-positive samples. The study included a total of 1194 samples, including 536 samples from FIV-infected cats, based on WB analysis.

## 2. Materials and Methods

### 2.1. Serum Samples

Feline plasma and serum samples submitted to the Clinical Laboratory, Vetsuisse Faculty, University of Zurich, Switzerland, from the end of 1998 until the beginning of 2019, were available for this study. The samples had been taken by veterinary practitioners as part of a diagnostic workup and were sent to the laboratory for routine diagnostic purposes; only leftover samples were used, and no additional blood volume was collected for the current study. No ethical approval was necessary for this study in compliance with Swiss regulations [81].

### 2.2. FIV-TM ELISA

The FIV-TM ELISA was performed as previously described [53], with some modifications. Feline serum samples were diluted 1:4500 and tested in duplicate. A positive and negative control was run with each plate. For the positive control, pooled sera from experimentally FIV-infected cats were used for every run [82]. The negative control consisted of heat-inactivated bovine serum. The plate was then incubated for one hour at 37 °C and washed three times. Horseradish peroxidase-labeled goat anti-cat immunoglobulin G (H+L) (Jackson Immunoresearch Laboratories, West Grove, PA, USA) was used as a conjugate and was diluted 1:1000. The absorbance was measured by an ELISA plate reader (SPECTRAmax PLUS 384, Bucher Biotec AG, Basel, Switzerland) at 405/550 nm. The optical density (OD) values of the cat serum samples were expressed as percentages of the value obtained from the positive control sera. Results that were >50% in comparison with the positive control were classified as positive, whereas those that were <10% were classified as negative and those that were 10–50% were classified as ambiguous.

### 2.3. FIV WB

The WB was conducted as previously described using 500 ng of gradient-purified FIV Z2, which was propagated in feline specified-pathogen free lymphocyte cultures in the presence of recombinant interleukin-2 [53,70,74]. Feline serum samples were diluted 1:50 and incubated with WB strips overnight on a rocker platform at room temperature. A peroxidase-labeled goat anti-cat immunoglobulin G (H+L) conjugate (Jackson Immunoresearch Laboratories) was diluted 1:1000 and incubated for two hours; after a washing step, the substrate (4-chloronaphtol, HRP Color Development 4CN, Bio-Rad Laboratories, Hercules, CA, USA) was added. A positive (serum from a FIV-positive cat) and negative control (buffer only) were run for each blot. The WB was considered positive if two bands with a molecular weight of 15,000 (p15) and 24,000 (p24) Dalton, respectively, were recognizable on the blotting strip [74]. If both bands were absent, the sample was judged as WB-negative. Samples that yielded only one band, either p24 or p15, were judged as inconclusive. WB-positive and negative results, but not inconclusive results, were included in the statistical analysis.

### 2.4. FIV Point-of-Care Testing

Depending on the remaining sample volume, ELISA-negative and WB-positive samples were tested using two different point-of-care tests: WITNESS^R^ FIV (Zoetis, Delémont, Switzerland) and/or SNAP^TM^ FeLV Ag/FIV Ab Combo Plus (IDEXX Laboratories, Westbrook, ME, USA). The kit materials and samples were allowed to warm to room temperature before performing the test (SNAP^TM^) or were used directly (WITNESS^R^) according to the manufacturer’s recommendations, and the reading of the results was performed 10 min after the activation of the test.

### 2.5. FIV RT-PCR Analysis

ELISA-false negative (WB-positive) samples that underwent one or both POCT tests and that had enough remaining volume for additional tests (*n =* 59) were further analyzed by RT-PCR. Additionally, a few ELISA false-negative samples with too little volume for combined POCT and RT-PCR analyses (*n =* 6) underwent only RT-PCR to increase the chance of viral RNA confirmation in at least one discordant sample. Moreover, some samples from FIV-infected cats (ELISA- and WB-positive) were included as controls. Total nucleic acid was extracted from 100 µL of EDTA anticoagulated blood (1/65) using the MagNA^R^ Pure LC^R^ Total Nucleic Acid Isolation Kit (Roche Diagnostics GmbH, Mannheim, Germany) or RNA from 140 µL anticoagulated plasma or serum (64/65) with the QIAamp^R^ Viral RNA Kit (QIAGEN GmbH, Hilden, Germany). Notably, prior to nucleic acid extraction, the samples had undergone an unknown number of freeze–thaw cycles, and some of them had been stored for up to 20 years at −20 °C. Negative controls consisting of 100 or 140 µL of phosphate buffered saline were prepared with each extraction batch to monitor cross-contamination.

Nucleic acid samples were analyzed by real-time RT-PCR that allowed the amplification of various FIV isolates from clades A and B as described previously [58,60], with the following modifications: the final reaction volume of 25 µL contained 800 nM of each primer (FIV551f/FIV571r), 160 nM of the fluorogenic probe (FIV581p), 12.5 µL 2× RT qPCR Mastermix and 0.125 µL of a master mix containing 6.25 U Euroscript reverse transcriptase and 2.5 U RNase inhibitor (One Step RT qPCR MasterMix Plus Low ROX, Eurogentec Headquarters, Seraing, Belgium). The cycling conditions were as follows: 30 min at 48 °C for reverse transcription, followed by an initial denaturation for 10 min at 95 °C and 45 amplification cycles at 95 °C for 15 s and 60 °C for 1 min as previously described by using a 7500 Fast Real-Time PCR System (ThermoFisher Scientific, Zug, CH) [60]. A second real-time RT-PCR was performed according to a modified version of the method used by Wang et al. [80], which used 900 nM of both the upstream (FIV_gag_upstr: 5′- ATG GGG AAY GGA CAG GGG CGA GA-3′) and downstream (FIV_gag_downstr: 5′- TCT GGT ATR TCA CCA GGT TCT CGT CCT GTA-3′) primers and 250 nM fluorogenic probe (FIV_gag_F2ABCEmIM 5′-FAM-TGG CCA TWA ARA (iQ500)GAT GYA GTA ATG TTG CTG TAG G-BHQ1-3′), 0.625 µL (40 U/µL) RNasin^R^ Plus (Promega, Madison, WI, USA), 12.5 µL 2× RT qPCR Reaction Mix, 0.5 µL Superscript^TM^ III RT/Platinum^R^ Taq mixture and 0.05 µL ROX (all from the Superscript^TM^ III Platinum^TM^ One-Step qRT-PCR kit, ThermoFisher Scientific, Carlsbad, CA, USA) in a final volume of 25 µL. The reaction mix underwent reverse transcription at 50 °C for 15 min, denaturation and activation for 2 min at 95 °C and 45 cycles of 95 °C for 15 s and 60 °C for 30 s. In addition, the nucleic acid samples were tested using a commercially available real-time RT-PCR kit (FTvet Feline Anaemia I, Fast Track Diagnostics, Esch-sur-Alzette, Luxembourg). Finally, a seminested RT-PCR was performed that amplified a 470 bp-long sequence from the FIV clade A and B *gag* gene using primers previously described [79,83] with a concentration of 200 nM of each primer in the first round and 1 µM of each primer in the second round. The final reaction volume was 25 µL in both rounds; 12.5 µL 2× Reaction Mix, 1 µL Superscript^R^ polymerase (Superscript^R^ III One-Step RT-PCR with Platinum^R^ Taq Kit, ThermoFisher Scientific) and 0.31 µL (40 U/µL) RNasin^R^ Plus (Promega) were used in the first round, whereas 2.5 µL 10× buffer, 1.5 µL MgCl_2_, 1.25 µL (5 U/µL) Taq DNA Polymerase (Sigma-Aldrich, St. Louis, MO, USA) and 0.5 µL dNTPs (ThermoFisher Scientific, Vilnius, Lithuania) were used in the second round. Briefly, a reverse transcription step was performed for 30 min at 55 °C, followed by the first round of PCR, which consisted of 2 min at 94 °C, 40 cycles of 15 s at 94 °C, 1 min at 53 °C and 1 min at 73 °C, and a final step of 5 min at 68 °C. Then, for the second round, 5 µL of the first round PCR product was used with the following cycling conditions: five minutes at 95 °C, followed by 40 cycles of 30 s at 95 °C, 30 s at 58 °C and 1 min at 72 °C and a final elongation step of 10 min at 72 °C. The PCR products from the second round of PCR were analyzed by agarose gel electrophoresis (2%). All bands of the expected length were purified (QIAQuick^R^ Gel Extraction Kit, QIAGEN GmbH) and submitted for sequencing (Microsynth AG, Balgach, Switzerland). The sequences were edited and assembled using Geneious^R^ 11.1.5 software (Biomatters, Ltd., Auckland, New Zealand). Using a basic local alignment search tool (NCBI: https://blast.ncbi.nlm.nih.gov/Blast.cgi), we aimed to find similarities between the isolated sequences and published FIV sequences.

### 2.6. Diagnostic Sensitivity and Specificity of FIV-TM ELISA

WB has been considered the gold standard to accurately identify FIV positive and negative samples. The diagnostic sensitivity, specificity and efficiency of FIV-TM ELISA were calculated.

### 2.7. Statistics

The data were compiled in Microsoft Excel 2016 and analyzed using GraphPad Prism 8 (Version 8.1.0; GraphPad Prism Software, La Jolla, CA, USA). The frequencies were compared using Fisher’s exact test (p_F_). The ages of the cats in different groups were compared using the Mann–Whitney U-test (p_MWU_). *p*-values < 0.05 were considered statistically significant.

## 3. Results

### 3.1. Sample Characteristics and Results of FIV WB Testing

Of the 1194 cats included in the study, ages were known for 700 cats (59%), and the median age was 5.0 years (Table 1). The sex of 712 (60%) cats was known: 192/1194 (16%) and 289/1194 (24%) were intact males or castrated males, respectively; 104/1194 (9%) and 127/1194 (11%) were intact or spayed females, respectively (Table 1). The samples were obtained from Switzerland (641/1194; 54%), Germany (475/1194; 40%), France (53/1194; 4%), Austria (5/1194; 0.4%), Finland (9/1194; 0.8%), and Italy (2/1194; 0.2%). For nine samples, the country of origin was unknown.

### 3.2. Comparison of WB-Negative and WB-Positive Cats

The results from the FIV WB analyses were used as the gold standard for the determination of FIV status in cats [53,70,74]. Of the 1194 tested samples, 536 were FIV WB-positive and 411 were FIV WB-negative, and 247 had inconclusive results because they produced only one band in WB (Table 2).

Of the 536 WB-positive cats, the age of 289 was known; the median age of the WB-positive cats was 6.0 years (Table 1). Seventeen cats were 0.5 years old or younger; the FIV-positive results in these cats could be the result of maternal antibodies. The sex of 335 WB-positive cats was known: the positive samples originated from 91 (27%) intact males, 172 (51%) neutered males, 34 (10%) intact females, and 38 (11%) spayed females (Table 1). Most of the WB-positive samples came from Switzerland (*n =* 261) and Germany (*n =* 253); only a few samples were sent from other countries (Table 1).

Of the 411 WB-negative cats, the age was known for 255 cats: the median age of the WB-negative cats was 4.0 years (Table 1). Information about the sex was available for 240 cats: there were 65 intact male (27%), 64 neutered male (27%), 44 intact female (18%), and 67 spayed female cats (28%). WB-negative samples were sent from Switzerland (*n =* 235), Germany (*n =* 134), France (*n =* 30), Austria (*n* = 3), Finland (*n* = 5), and Italy (*n* = 1); for three samples, the origin was unknown.

The samples included in this study were not collected randomly but originated mainly from cats who were clinically suspected of FIV infection; nonetheless, some basic descriptive analyses were performed. FIV WB-positive cats were significantly more likely to be male, either intact or castrated (263/335; 79%) than FIV WB-negative cats (129/240; 54%; p_F_ < 0.0001) (Table 1). The median age differed significantly between WB-positive (median age 6.0 years) and WB-negative cats (median age 4.0 years) (p_MWU_ = 0.0360). FIV WB-positive samples originated less frequently from Switzerland (261/530; 49%) than WB-negative samples (235/408; 58%; p_F_ = 0.0122).

### 3.3. Confirmation of FIV-TM ELISA Results Using FIV WB

Most of the WB-negative samples (375/411; 91%) were FIV-TM ELISA-negative or showed an ambiguous result according to ELISA (25/411; 6%; Table 2). It is recommended that ambiguous FIV-TM ELISA results be directly retested by WB. Therefore, they do not pose an imminent problem in terms of the diagnosis of FIV infection (false positive/false negative) and were not further analyzed in this study. However, 3% of the WB-negative samples (11/411) were found to be false-positive according to FIV-TM ELISA.

Of the 536 samples that were FIV WB-positive, 441 (82%) were also TM ELISA-positive, and 25 (5%) revealed an ambiguous result. Remarkably, 70 WB-positive samples (13%) were negative according to TM ELISA (Table 2).

The diagnostic sensitivity (true positives/all positives) of FIV-TM ELISA was 82% (441/536); the diagnostic specificity (true negatives/all negatives) was 91% (375/411), and the diagnostic efficiency (correct tests/all tests) was 86% (816/947).

### 3.4. False ELISA-Positive Samples (WB-Negative and FIV-TM ELISA-Positive)

Overall, 11 cats were false-positive according to FIV-TM ELISA (WB-negative but ELISA-positive; Table 3). For eight samples, a sufficient volume was available to test them also using one or both of the POCT. Two samples tested negative in both POCT and six were positive in at least one or both of the POCT performed (Table 3). For three samples, no POCT could be performed. POCT-negative samples had OD values in the FIV-TM ELISA in the low positive range, according to our definition (positive >50% of the positive control). POCT-positive samples had, with one exception, OD values >100% of the positive control in the FIV-TM ELISA.

### 3.5. False ELISA-Negative Results (WB-Positive and FIV-TM ELISA-Negative)

The characteristics of the cats with false-negative ELISA results (WB-positive but ELISA-negative) are summarized in Table 1, and the data for each cat are given in Table 4. Such samples originated more frequently from male cats (29/70; 41%) than from female cats (16/70; 23%). The samples were sent mainly from Switzerland (38/70; 54%) or Germany (27/70; 39%); five samples were from France (Table 1). The median age of cats with false-negative ELISA results (5.0 years; Table 1) was lower than that of cats with FIV WB- and ELISA-positive results (6.0 years; Table 1), and a minimum age of two months was observed in both groups. The oldest cat with a false-negative ELISA result was 16 years old, while among the WB- and ELISA-positive cats, 18 years was the maximum age. For several sampling years, there were only one or two false-negative cases per year (<10% of all WB-positive samples sent in the respective year), while there were four discordant cases each in 2004 and 2006 and fourteen in 2005 (17%/41%/31% of all WB-positive samples in 2004/2005/2006, respectively). The discordant samples represented 15% of all WB-positive samples in 2014 (*n* = 3), 29% in 2015 (*n* = 12), 24% in 2016 (*n* = 11), and 19% in 2017 (*n* = 8) (Table 1, Table 4).

### 3.6. Further FIV Testing of False ELISA-Negative Samples

Of the 70 samples with false-negative ELISA results (WB-positive and TM ELISA-negative), 64 could be tested with at least one of the two POCT, WITNESS^R^ or SNAP^TM^. Sixty samples had enough sample volume to perform the first POCT (WITNESS^R^), and 48 of these also had enough volume to perform the second POCT (SNAP^TM^). Four additional samples were only tested in the SNAP^TM^ test (in total, 52 samples were SNAP^TM^-tested). In the WITNESS^R^ test, one sample (2%) tested clearly positive (Table 4). One sample (sample ID 00005100) showed a band only after the regular reading time at 10 min had passed, and 58/60 (97%) were clearly negative. Of the 52 samples used in the SNAP^TM^ test, five samples (10%) were positive and 47/52 (90%) were negative (Table 4). Both samples that tested positive or late positive in the WITNESS^R^ test were also positive in the SNAP^TM^ test. The SNAP^TM^ test detected two additional samples that were negative in the WITNESS^R^ test and one that was not tested with the WITNESS^R^ test. While most of the false ELISA-negative samples had very low ODs according to FIV-TM ELISA (0–3%), one of the samples that was double positive in the two POCT and one sample that was only tested with the SNAP^TM^ test had ODs of 9.0% and 8.0%, respectively, which were just barely below the cut-off value for ambiguous samples (Table 4). An additional sample that was negative in both POCT showed a result of 9.9% (sample ID 00012612), which was just at the cut-off value of 10% for FIV-TM ELISA. The two SNAP^TM^/WITNESS^R^ double positive samples and the positive sample that was tested only with the SNAP^TM^ test were sent from France and Switzerland, and the two samples that were detected solely by the SNAP^TM^ test were from Germany and Switzerland.

Enough sample volume remained for 65 of the 70 discordant samples for nucleic acid extraction and to perform FIV RT-PCR. In six of the 65 samples tested using RT-PCR, no POCT was performed because the remaining material was sufficient only for POCT or RT-PCR. To increase the chance of RT-PCR confirmation and subsequent sequencing in the discordant samples, it was decided to use RT-PCRs for these low volume samples. None of the 65 samples tested positive by a previously described FIV real-time RT-PCR that allowed the amplification of various FIV isolates from clades A and B [60] or by a modification of a RT-PCR method described by Wang [80]. Three samples tested weakly positive (CT-values: 38/39/40) according to a commercial real-time RT-PCR kit (FTvet Feline Anaemia I; Table 4). The seminested RT-PCR revealed a very weak band for one cat that could not be sequenced. Five ELISA- and WB-positive samples were also analyzed as controls using RT-PCR. The five samples had high OD values in the TM ELISA (>100%) and were positive in both POCT. All five samples were positive according to at least two different RT-PCR assays (Table 5). A BLAST search of the sequences demonstrated high similarities to deposited sequences from subtypes A and B.

### 3.7. Inconclusive WB Results

We found 247 samples with inconclusive WB results (Table 2). Of these samples, the majority showed antibodies against p24 (230/247, 93%); only a few had a p15 band (17/247; 7%). Of the 247 samples, 36 were ELISA-positive, of which 30 had OD values ranging from 100% to 440% of that of the positive control; 205 tested ELISA-negative and six had an ambiguous result in the FIV-TM ELISA (Table 2). Animals with inconclusive WB results are recommended to be retested two to three months later.

## 4. Discussion

In the present study, we investigated our hypothesis that the number of FIV infections in domestic cats that are undetectable by FIV-TM ELISA and FIV POCT screening assays has increased over the years. We have sporadically identified feline plasma and serum samples that were positive according to a confirmatory FIV WB test but negative according to a FIV-TM ELISA screening assay. This phenomenon had already been described during the development of the FIV-TM ELISA in 1995, but it only concerned four of 194 tested samples, and they all originated from cats that had lived outside of Switzerland [53]. We assumed that the number of discordant samples could potentially have risen due to an increased number of cats with a travel or import history, which might have resulted in the introduction of novel FIV isolates in the sample population. For some of the routine diagnostic cases with discordant results, it was confirmed by veterinarians or the owners that the cats had been imported. To further investigate our hypothesis, we systematically retested the available feline plasma and serum samples submitted to our laboratory over the last two decades (1998–2019) for FIV diagnosis or confirmation using FIV-TM ELISA and WB, which was considered the gold standard confirmation method.

The results of our study demonstrate an increased percentage of cats in Central Europe that tested FIV-positive by WB, but FIV infection in these cats was unrecognized by FIV-TM ELISA as well as some FIV POCT. In the earlier study mentioned above [53], the FIV-TM ELISA used herein had both a high diagnostic sensitivity and specificity of 98% and 97%, respectively, when using WB as the gold standard [53]. Only 2% of the 194 FIV WB-positive samples were negative according to the TM ELISA [53] compared to 70 out of 536 FIV WB-positive samples (13%) collected from August 1998 to February 2019 and analyzed in the current study. These results were found to indicate a reduced diagnostic sensitivity of FIV-TM ELISA of 82%, compared to 98% in 1995. In an attempt to corroborate our results regarding the reduced diagnostic sensitivity of FIV-TM ELISA, we also used two POCT for samples with ample remaining sample volumes. Remarkably, the two POCT did not recognize many of the discordant samples. This observation has serious diagnostic implications, since FIV-TM ELISA as well as FIV POCT are used as screening assays for FIV infection; they should have the highest sensitivity possible in order not to miss any FIV-infected cats during the primary diagnostic screening step [75].

In the earlier study in 1995, the four samples with discordant results originated from cats in countries other than Switzerland, and it was hypothesized that FIV-TM ELISA might be specific for certain variants of FIV [53,62,64]. It was assumed that for the routine testing of cats within Switzerland and in the absence of a travel or import history of the cat, the sensitivity of FIV-TM ELISA was sufficient. However, in the current study, more than half of the cats with discordant results (54%) had either presented to Swiss veterinarians or were represented by samples that had been sent to a Swiss laboratory before they were sent for confirmation to our laboratory. This does not necessarily imply that all these cats had lived in Switzerland; especially in regions close to the border, the country of the veterinarian was not necessarily the country of the origin of the cat. Therefore, a few of these cats with an FIV infection that was unrecognized by FIV-TM ELISA might also have lived abroad. In addition, the remaining 46% of cats with discordant results originated mainly from Germany, and some originated from France. Moreover, based on personal communication with a cat owner, we knew that at least one of the cats in Switzerland with a discordant FIV test result was originally imported from Greece. Based on earlier studies, FIV subtype A is the predominant subtype in Switzerland and Germany, while in southern Europe and Turkey, FIV subtype B is more common [16,20,24,29,32,34,35,36,37]. We were unable to further analyze the prevailing FIV subtypes in the cats with discordant results, since only a few were found to be positive using various RT-PCR assays. Only serum or plasma was available, so no provirus PCR, which requires anticoagulated whole blood, could be conducted; this test usually has a higher sensitivity for FIV infection [84]. Moreover, in the few RT-PCR positive samples, the viral RNA loads were too low to sequence the virus. Nonetheless, our data indicate that there is currently an increased number of FIV-infected cats in Switzerland as well as in Germany that harbor FIV isolates that induce antibodies that are unrecognized by the TM antigen used in FIV-TM ELISA and the two FIV POCT. Considering the increased travel of cats in Central Europe, particularly from the East and the South to the North and West of Europe, this might concern more European countries than just Switzerland and Germany.

TM is the immunodominant epitope in FIV that induces the earliest and strongest antibody response in cats experimentally infected with FIV [53,62,63,71,72]. Although the evolutionary rate of FIV is rather slow when compared to other lentiviruses [85] and seems to be dependent on the virus strain and the infection stage [86], it has been shown that the preferred genetic location for recombination is in the envelope (*env*) gene, which also encodes TM [10,25,61,87]. Additionally, low fidelity in the transcription process and a change in the clade distribution could have contributed to the increased variability of this protein. The appearance of new viral quasispecies in infected cats has been reported as being common [50,88]. During virus transmission, these quasispecies infect the new host and undergo further mutation, hence broadening the genetic diversity [89]. The importance of the export of domestic cats with virus strains distinct from those locally predominant might result in a change in virus strain distribution or even the spread of regionally clustered subtypes to new areas [16,17,26,30,32,33,34,42]. Taking into account exchanges between zoos, interhost transmission over wide ranges and behavioral changes caused by human expansion, nondomestic felids are also a potential source [90,91,92,93,94,95]. Therefore, not only will intrasubtype recombination occur, but the development of new subtypes arising from recombination between strains from distinct clusters will become more likely [16,27,31,41,47].

TM is encoded in the variable *env* gene and is mainly responsible for the host antibody response [61,62,63]. Mutations in the nucleotide sequence can lead to structural changes and result in antibodies unrecognizable by common diagnostic tests [53,61,62,63,64]. In contrast, the capsid protein p24 and the matrix protein p15 are encoded in the *gag* region and are considered to be highly conserved [25,61,96]. Therefore, antibodies to these epitopes, if present, should be more consistently recognized, and it has been suggested that they be included in serological testing for FIV [53,72]. Both *gag* proteins are used in the WB analysis as well as in one of the included POCT, the SNAP^TM^ test, which is an ELISA-based test that also includes TM [53,56,70,73,74,97]. The second POCT included in this study, the WITNESS^R^ test, is an immune chromatography-based assay that detects antibodies directed against TM but not against *gag* proteins, similarly to the FIV-TM ELISA [56,73,98] used herein. Both POCT showed a low sensitivity in the present study when testing samples from cats with discordant results. Only five of the tested cats with discordant results (60 for the WITNESS^R^ test and 52 for the SNAP^TM^ test) had a positive POCT result; two of the POCT-positive results could be confirmed using RT-PCR. A third POCT double positive result was found in a kitten that was 3 months of age. In a follow-up sample collected at the age of 6 months, the kitten was shown to be FIV-negative by WB; thus, the initial POCT-positive results at 3 months were most likely due to maternal antibodies and the RT-PCR result was truly negative, since no viral RNA was present. The fourth SNAP^TM^ POCT-positive cat was negative according to the WITNESS^R^ POCT test as well as all RT-PCR methods employed. Because the age of this cat was not known and the SNAP^TM^ signal was weak, the presence of maternal antibodies was not fully excludable. Two additional FIV WB-positive samples, which were found to be negative by FIV-TM ELISA and both POCT, were weakly positive according to either Fasttrack RT-PCR (CT-value of 37.8) or seminested RT-PCR (very weak band). Thus, FIV infection could be confirmed using a different methodology to detect the virus itself instead of antibodies directed against the virus. The possibility cannot be completely excluded that these samples were collected during a very early infection before ample titers of antibodies had developed; the WB method might be somewhat more sensitive to this kind of early infection. However, we usually do not see both bands, p15 and p24, in the early phase of infection [52,53,72], which was the case in these two samples. Overall, 247 samples in the present study showed inconclusive WB results with only the p24 or p15 band. Some of these samples might have originated from cats in an early FIV infection. In order to distinguish the latter from unspecific reactions, animals with inconclusive results according to WB are recommended to be retested two to three months later for a definite diagnosis.

A limitation of this study is that the samples had been stored at −20 °C for up to 20 years. However, antibodies are not sensitive to long-term storage at −20 °C, and repeated freeze–thaw cycles have a minimal detrimental effect [99]. In contrast, viral RNA is very sensitive to degradation and might thus have been lost during storage [100]. This might have contributed to the many negative RT-PCR results in the discordant samples, in addition to the expected low viral loads in many FIV-infected cats and sequence variation in the FIV genome, which might have led to a lack of recognition by the oligonucleotides used in the RT-PCR assays. All RT-PCR-positive samples showed very low viral loads that were too low for sequencing. Because of the long storage of many of the samples, repeated freeze–thawing cycles, the limited sample volume and uncertainty concerning the sterility of the samples, we did not consider virus isolation from cell culture. Since not all discordant samples could be tested with all methods available in this study, it is difficult to compare the results of the different tests. However, overall, the SNAP^TM^ POCT test seemed to have recognized more of the discordant samples than the WITNESS^R^ POCT test. Two SNAP^TM^ POCT-positive samples were negative according to the WITNESS^R^ POCT. It is not quite clear why the SNAP^TM^ POCT, which identifies the gp40, p15, and p24 antigens, did not detect all WB-positive samples, as antibodies against p15 and p24, two highly conserved antigens, were obviously present in the samples, since they had been recognized by WB. One argument for the enhanced sensitivity of WB compared to that of the SNAP^TM^ test could be that the antigen concentration per strip and the accessibility of the antigen could be higher than that for the SNAP^TM^ test, in which three antigens share one reaction field. We have no information on the exact antigens used in the different tests and the conformation and presentation of the antigens. Differences in the specificities of the antigens in the different tests, the import of new viruses and mutations, and the recombination of viruses and resulting changes in their antigens and the specificity of induced antibodies could have contributed to the discordant test results. Moreover, the sensitivity of the different tests could have played a role. This was obvious in one sample (sample ID 00005100) that was clearly positive in the SNAP^TM^ POCT but negative in the WITNESS^R^ POCT at the normal reading time point of 10 min and became weakly positive thereafter. Moreover, one sample (sample ID 00012612) was just at the cut-off point for a negative result (10%) for FIV-TM ELISA (9.9%). This sample was negative according to both the SNAP^TM^ and WITNESS^R^ POCT. As the results of all methods except FIV-TM ELISA are determined by visual inspection, positive samples could be falsely interpreted as negative if the colorimetric signal was not strong. It has been reported before that the sensitivities of the POCT used herein differ depending on the respective geographic location and the study cohort tested from 89 to 100% for the SNAP^TM^ test and 93.8 to 100% for the WITNESS^R^ test [68,73,98,101,102,103].

The decreased sensitivity of FIV-TM ELISA described over the last twenty years was found based on a comparison with a study from 1995 [53]. The investigated samples in the current study were influenced by the number of samples submitted to our laboratory, the awareness of veterinarians of FIV infection in general and the necessity to test for FIV and to confirm POCT results as well as the geographic distribution of submitting customers. This has led to a high number of discordant samples, with up to 14 discordant samples per year in some years and little or no discordant samples in other years. Therefore, we were unable to determine whether the decrease in sensitivity occurred gradually over time and whether it poses an increasing problem, but overall it seems to be of significant relevance. In our study, the number of false-negative samples (*n* = 70) greatly surpassed the number of false-positive samples (*n* = 11); the issue with false-positive results has been generally recognized in countries with a low FIV prevalence [56,76,78]. The majority of false-positive samples according to FIV-TM ELISA in our study were also false-positives when tested with the POCT, emphasizing the need for confirmation using WB to obtain a definitive FIV diagnosis.

Finally, the discordant samples could also have been falsely WB-positive. We have chosen and used WB as the gold standard for detecting FIV infection for many years. In countries where no FIV vaccine is available, which is now the case for the US and has always been the case in Europe, WB is accepted as the gold standard for FIV diagnosis and for the confirmation of ambiguous and positive samples [56,65,70,73,74,75,76,104]. An alternative method for confirmation of presence or absence of FIV infection is virus isolation by cell culture. However, this method requires the purification and culture of fresh peripheral blood mononuclear cells from the cat under investigation ideally with cells from an uninfected cat; the method is laborious, expensive and time-consuming and is only offered in a few specialized laboratories [55,56,65,67,105]. No lymphocyte-containing fresh whole blood samples were available for this study, precluding this option. In agreement with good laboratory practice, we always run negative and positive controls with each blot to control for false-positive or false-negative results [106]. Some of the discordant samples in this study could be confirmed to be positive either using one of the two POCT employed in this study or RT-PCR, even though some of the samples had been stored for a long time. Finally, there is evidence that WB is more robust for the detection of antibodies against a variety of felid lentiviruses: WB has been positively used in wild felid species, such as lions, cheetahs, leopards, or Geoffroy’s cats [3,5,6,9,53,107,108,109,110]. For these reasons, we are quite confident that the WB-positive samples are from cats with true FIV infections.

## 5. Conclusions

In conclusion, FIV screening solely relying on antibodies directed against a single TM protein seems to be no longer adequate in geographic areas where cats with imported and new viruses must be expected. However, a POCT (SNAP^TM^ FIV/FeLV Combo) using additional FIV antigens besides the immunodominant TM antigen did not recognize all the presumptively FIV-positive cats. Since ELISA and POCT are used for screening purposes, the inability to recognize an increasing number of FIV-infected cats poses a serious problem. Currently, it is recommended to confirm any ELISA and POCT ambiguous or positive results in countries with a low FIV prevalence, since the positive predictive value of a positive ELISA and POCT result is low under these circumstances [56,76,78]. However, in light of our results, we now additionally recommend that any cat with a high suspicion of FIV infection and a negative FIV screening test be further investigated using WB for the purposes of confirmation to exclude false-negative results. Future prospective studies should aim to characterize in-depth fresh samples from cats with discordant results to identify the underlying viruses to further improve the laboratory diagnosis of FIV infection.

## Figures and Tables

**Table 1:** Sample characteristics: Sex, age, and origin of the cats that underwent FIV WB and ELISA testing.

	All Samples	WB-Negative/Inconclusive Samples ^1^	WB-Positive Samples ^1^	WB-Positive ^1^, ELISA-Positive Samples	WB-Positive ^1^ Samples with Ambiguous ELISA Results	WB-Positive ^1^, ELISA-Negative Samples
**Total**	1194	411/247	536	441	25	70
**Sex**						
- m	192	65/36	91	74	2	15
- mc	289	64/53	172	155	3	14
- f	104	44/26	34	24	0	10
- fs	127	67/22	38	31	1	6
- unk	482	171/110	201	157	19	25
**Age** (y)						
- < 6 months ^2^	46	23/12	11	6	1	4
- 6 months to < 2 y	113	49/25	39	29	0	10
- 2 to < 6 y	210	70/48	92	74	4	14
- 6 to < 10 y	151	44/31	76	63	0	13
- 10 to < 14 y	125	45/25	55	50	0	5
- 14 to < 18 y	47	18/14	15	8	0	7
- 18 to < 23 y	8	6/1	1	1	0	0
- unk	494	156/91	247	210	20	17
**Origin**						
- CH	641	235/145	261	216	7	38
- DE	475	134/88	253	215	11	27
- FR	53	30/9	14	2	7	5
- AT	5	3/2	0	0	0	0
- FI	9	5/3	1	1	0	0
- IT	2	1/0	1	1	0	0
- unk	9	3/0	6	6	0	0
**Collection** (y)						
1998–2003	252	99/28	125	117	3	5
2004–2008	211	44/53	114	83	7	24
2009–2013	276	124/56	96	84	8	4
2014–2019	455	144/110	201	157	7	37

^1^ FIV-negative and FIV-positive were defined according to the gold standard test, WB; samples producing one band in WB are “inconclusive”; ^2^ maternal antibodies are possible in WB-positive cats known to be younger than 6 months of age; unk = unknown; m = male, mc = castrated male, f = female, fs = spayed female; y = years; CH = Switzerland, DE = Germany, FR = France, AT = Austria, FI = Finland, IT = Italy.

**Table 2:** Comparison of FIV WB and FIV ELISA results.

	WB-Positive	WB-Negative	WB-Inconclusive ^2^	Total
**ELISA-Positive** (OD <50% of pc ^1^)	441	11	36	488
**ELISA-Negative** (<10% of pc)	70	375	205	650
**Ambiguous Result** (10–50% of pc)	25	25	6	56
**Total**	536	411	247	1194

^1^ pc = positive control run with every ELISA; **^2^** WB-inconclusive samples result in only one band, p24 or p15.

**Table 3:** Characteristics of the 11 false ELISA-positive samples (FIV WB-Negative and FIV-TM ELISA-Positive).

Sample ID	Age of Cat (Years)	Sex of Cat	Year of Sample Collection	Origin of Sample (Country)	ELISA (% of pc)	SNAP^TM^ POCT	WITNESS^R^ POCT
1322	15	m	1999	CH	64.0	neg	neg
1383	unk	m	2000	CH	60.0	nt	nt
1436	unk	unk	2000	CH	133.7	nt	pos
1607	9	m	2004	DE	149.4	pos	pos
1758	14	fs	2007	DE	70.7	pos	pos
1892	3	mc	2009	CH	75.0	neg	neg
1995	6	f	2011	CH	107.9	pos	pos
2021	12	fs	2011	CH	51.0	nt	nt
2022	10	unk	2011	CH	51.0	nt	nt
2023	0.4	m	2011	CH	108.8	pos	pos
41673826	7	fs	2017	CH	102.5	nt	pos

unk = unknown; m = male, f = female, mc = castrated male, fs = spayed female; nt = not tested since no more material was available; neg = negative, pos = positive; pc = positive control; CH = Switzerland, DE = Germany.

**Table 4:** Characteristics of the 70 false ELISA-negative samples (FIV WB-positive and FIV-TM ELISA-negative).

Sample ID	Age of Cat (Years)	Sex of Cat	Year of Sample Collection	Origin of Sample (Country)	SNAP^TM^ POCT	WITNESS^R^ POCT	ELISA (% of pc)	RT-PCR
1343	unk	unk	1999	CH	neg	neg	1.5	neg
1359	1	m	1999	CH	neg	neg	1.7	neg
1537	4	mc	2002	DE	neg	neg	0.0	neg
1554	8	fs	2003	CH	neg	neg	3.1	neg
1574	7	mc	2003	CH	neg	neg	1.3	neg
1599	5	f	2004	DE	neg	neg	0.0	neg
1621	unk	unk	2004	CH	nt	neg	0.0	neg
1633	3	fs	2004	DE	neg	neg	3.3	neg
1634	8	f	2004	DE	nt	nt	1.5	neg
1648	unk	unk	2005	CH	nt	neg	0.0	neg
1656	1	m	2005	DE	nt	nt	0.0	neg
1666	unk	unk	2005	CH	neg	neg	0.0	neg
1674	0.2 ^1^	m	2005	CH	neg	neg	0.0	neg
1678	2	mc	2005	DE	nt	nt	0.0	neg
1679	0.25 ^1^	m	2005	DE	nt	neg	0.0	nt
1683	0.5	m	2005	DE	neg	neg	3.0	neg
1686	unk	m	2005	DE	neg	neg	0.0	neg
1690	3	unk	2005	CH	neg	neg	0.6	neg
1691	13	mc	2005	DE	nt	nt	0.0	neg
1692	unk	unk	2005	CH	nt	nt	0.0	neg
1698	8	m	2005	DE	nt	neg	0.0	neg
1699	8	mc	2005	DE	neg	nt	0.0	nt
1700	5	mc	2005	DE	neg	neg	0.0	neg
1701	14	fs	2006	DE	neg	nt	0.0	nt
1703	13	mc	2006	DE	nt	nt	0.0	neg
1704	2	mc	2006	CH	neg	neg	0.0	neg
1710	3	mc	2006	DE	neg	nt	0.0	neg
1781	6	f	2007	DE	neg	neg	0.0	**pos ^2^**
1812	2	f	2008	DE	neg	neg	2.5	neg
1886	unk	unk	2009	FR	**pos**	**pos**	**9.0**	**pos ^2^**
1922	unk	unk	2010	DE	nt	neg	0.0	neg
1925	unk	unk	2010	FR	nt	neg	0.0	neg
00005100	0.25 ^1^	m	2013	CH	**pos**	**pos** ^4^	**6.0**	nt
00006038	unk	unk	2014	DE	neg	neg	0.0	neg
00007702	unk	unk	2014	DE	neg	neg	0.0	neg
00007792	0.5	m	2014	DE	nt	neg	1.6	neg
00008569	9	mc	2015	DE	neg	neg	0.0	neg
00009944	6	mc	2015	CH	neg	neg	0.0	neg
00009955	unk	unk	2015	FR	**pos**	nt	**8.0**	nt
00010012	1	m	2015	CH	neg	neg	0.0	neg
00010489	0.6	mc	2015	CH	nt	neg	0.9	neg
00010545	0.3^1^	f	2015	CH	neg	neg	2.0	neg
41385749	10	unk	2015	CH	neg	neg	0.0	**pos ^3^**
41386411	12	unk	2015	CH	neg	neg	0.2	neg
41387194	2	unk	2015	CH	neg	neg	0.4	neg
41387409	10	unk	2015	CH	neg	neg	0.2	neg
41388050	16	unk	2015	CH	neg	neg	0.3	neg
41388399	6	unk	2015	CH	neg	neg	0.1	neg
41392409	16	unk	2016	CH	neg	neg	1.5	neg
00010770	unk	f	2016	DE	neg	neg	0.0	neg
41397627	8	unk	2016	CH	neg	neg	0.1	neg
41398102	6	unk	2016	CH	neg	neg	0.2	neg
41404918	14	m	2016	CH	neg	neg	2.0	neg
41406670	1	m	2016	CH	neg	neg	0.0	neg
41406844	0.5	f	2016	CH	neg	neg	0.2	neg
00011456	3	fs	2016	CH	neg	neg	1.1	neg
41561426	16	f	2016	CH	neg	neg	0.3	neg
41577198	14	m	2016	CH	neg	neg	0.3	neg
00012612	4	fs	2016	CH	neg	neg	**9.9**	neg
00013274	unk	unk	2017	FR	nt	neg	0.0	neg
00013276	unk	unk	2017	FR	nt	neg	4.1	neg
00013381	7	mc	2017	CH	neg	neg	1.0	neg
00014059	0.8	f	2017	DE	neg	neg	3.0	neg
00014227	unk	unk	2017	DE	neg	neg	1.6	neg
00014598	2	m	2017	CH	nt	neg	0.0	neg
41663426	16	mc	2017	CH	neg	neg	0.0	neg
41669714	7	m	2017	CH	**pos**	neg	3.6	**pos ^2^**
00016295	unk	unk	2018	DE	**pos**	neg	5.3	neg
00018980	0.7	fs	2019	CH	neg	neg	5.0	neg
51028174	2	f	2019	CH	nt	neg	**8.0**	neg

^1^ maternal antibody presence possible; ^2^ FTvet Feline Anaemia I-positive, CT-value = 38–40; ^3^ seminested RT-PCR: weakly positive [79,83]; ^4^ only positive after the regular reading time at 10 min; unk = unknown, m = male, f = female, mc = castrated male, fs = spayed female; nt = not tested since no more material was available; neg = negative, pos = positive; pc = positive control; CH = Switzerland, DE = Germany, FR = France.

**Table 5:** Characteristics of five ELISA- and WB-positive control samples.

Sample ID	Age of Cat (Years)	Sex of Cat	Year of Sample Collection	Origin of Sample (Country)	ELISA (% of pc)	SNAP^TM^ POCT	WITNESS^R^ POCT	RT-PCR
1600	2	mc	2004	DE	111.0	pos	pos	pos ^1,2^
1622	2	mc	2004	DE	132.8	pos	pos	pos ^1,2^
1637	2	mc	2004	CH	182.3	pos	pos	pos ^1,2^
00015713	8	mc	2017	CH	179.2	pos	pos	pos ^1,2^
00016945	12	fs	2018	CH	265.5	pos	pos	pos ^1,2,3^

^1^ FTvet Feline Anaemia I-positive, CT-value = 30–40; ^2^ seminested RT-PCR-positive [79,83]; ^3^ RT-PCR-positive [60], CT-value = 33; mc = castrated male, fs = spayed female; pos = positive; pc = positive control; CH = Switzerland, DE = Germany.
